# BK Channels Are Activated by Functional Coupling With L-Type Ca^2+^ Channels in Cricket Myocytes

**DOI:** 10.3389/finsc.2021.662414

**Published:** 2021-04-21

**Authors:** Tomohiro Numata, Kaori Sato-Numata, Masami Yoshino

**Affiliations:** ^1^Department of Physiology, School of Medicine, Fukuoka University, Fukuoka, Japan; ^2^Department of Biology, Tokyo Gakugei University, Tokyo, Japan; ^3^Japan Society for the Promotion of Science, Tokyo, Japan

**Keywords:** BK channel, patch clamp, cricket, oviduct, myocyte, functional coupling

## Abstract

Large-conductance calcium (Ca^2+^)-activated potassium (K^+^) (BK) channel activation is important for feedback control of Ca^2+^ influx and cell excitability during spontaneous muscle contraction. To characterize endogenously expressed BK channels and evaluate the functional relevance of Ca^2+^ sources leading to BK activity, patch-clamp electrophysiology was performed on cricket oviduct myocytes to obtain single-channel recordings. The single-channel conductance of BK channels was 120 pS, with increased activity resulting from membrane depolarization or increased intracellular Ca^2+^ concentration. Extracellular application of tetraethylammonium (TEA) and iberiotoxin (IbTX) suppressed single-channel current amplitude. These results indicate that BK channels are endogenously expressed in cricket oviduct myocytes. Ca^2+^ release from internal Ca^2+^ stores and Ca^2+^ influx via the plasma membrane, which affect BK activity, were investigated. Extracellular Ca^2+^ removal nullified BK activity. Administration of ryanodine and caffeine reduced BK activity. Administration of L-type Ca^2+^ channel activity regulators (Bay K 8644 and nifedipine) increased and decreased BK activity, respectively. Finally, the proximity between the L-type Ca^2+^ channel and BK was investigated. Administration of Bay K 8644 to the microscopic area within the pipette increased BK activity. However, this increase was not observed at a sustained depolarizing potential. These results show that BK channels are endogenously expressed in cricket oviduct myocytes and that BK activity is regulated by L-type Ca^2+^ channel activity and Ca^2+^ release from Ca^2+^ stores. Together, these results show that functional coupling between L-type Ca^2+^ and BK channels may underlie the molecular basis of spontaneous rhythmic contraction.

## Introduction

The calcium (Ca^2+^)-activated potassium (K^+^) (BK) channel has a large single-channel conductance (~100–300 pS) (or MaxiK), hence the nickname “Big K” (1, 2). The BK channel α-subunit plays a central role in channel function. BK channel α-subunit homologs are found in a wide variety of organisms, from invertebrates, such as *Drosophila* and *Caenorhabditis elegans*, to vertebrates, including fish, mice, and rats (1–4). BK channels are expressed in nerves and muscles, and in endocrine, cardiovascular, digestive, urinary, and reproductive organs (1, 2, 5, 6). BK channel activity is regulated by membrane depolarization and increased intracellular Ca^2+^ concentration ([Ca^2+^]_i_). BK channel activation results in membrane repolarization and voltage-gated Ca^2+^ channel closure, reducing Ca^2+^ entry into cells. BK channels primarily function as negative-feedback regulators of membrane potential and [Ca^2+^]_i_, which are important in many physiological processes. BK activity is involved in action potential intervals, duration, firing frequency, neurotransmitter release, endocrine secretion, smooth muscle contraction, and control of epithelial cell potassium release in nerves (6–10). At the tissue level, the BK channels are functionally involved in movement disorders, circadian rhythms, learning and memory, hearing, vision, cardiovascular function, airway control, urination, glucose homeostasis, renal homeostasis, digestive function, immunity, body weight, pain, and bone remodeling. Consistent with their physiological importance, BK channel mutation and dysfunction can lead to epilepsy, Alzheimer's disease, noise-induced hearing loss, ataxia, congenital visceral malformations, hypertension, urinary incontinence, diabetes mellitus, cancer, and asthma (6–10). In vertebrates, the importance of BK channels at levels from the molecular to the organism has been comprehensively demonstrated.

A reductionist approach, using various simple forms of invertebrate behavior modification with simple physiological response systems, can provide biological insights into ion channels. Using this approach, channelopathies were identified from an extensive collection of *Drosophila* mutations (11). Additionally, the basis of ion channel function was identified from accurate action potential measurements using the giant squid axon (12) and was shown to be involved in *Aprian* learning (13), and the role of ion channels was identified by integrated-approach physiology and behavioral genetics in *C. elegans* (14). Despite the usefulness of invertebrates as model organisms, there is little information about their endogenous ion channel expression.

Membrane proteins can be organized by functional coupling with other proteins. Our understanding of systematic physiological reactions has been enhanced by focusing on the relationship between effector molecules and targets. BK channel activity may be regulated by a network of proteins involved in [Ca^2+^]_i_ regulation. Indeed, many reports suggest that influx Ca^2+^ through different channel types leads to activation of Ca^2+^-activated channels. BK channels can be activated by Ca^2+^ influx via N-methyl-D-aspartate receptors (15) and voltage-gated N- (16, 17), L- (18, 19), N- (20), P/Q- (21), and R-type Ca^2+^ channels (22). The ryanodine receptor on the endoplasmic reticulum is another Ca^2+^ effector molecule that regulates membrane excitability by controlling BK channel activity with Ca^2+^-induced Ca^2+^ release (23, 24). Therefore, BK channels are less sensitive to [Ca^2+^]_i_ at the resting membrane potential (~10 μM) (9) and must be close to the Ca^2+^ source to function. The issue of proximity has become a hot topic for researchers investigating the effect of functional ion channel complex formation on membrane excitation (25–27).

Plasma membrane Ca^2+^ channels and ryanodine receptors are involved in spontaneous rhythmic contractions of the cricket lateral oviduct (28). Furthermore, muscle contractions in blood vessels and the bladder, which are accompanied by many Ca^2+^ vibrations, are generated by functional coupling between voltage-gated Ca^2+^ channels and BK channels (29, 30). However, the functional binding of BK channels to Ca^2+^ sources in myogenic spontaneous rhythmic contractions has not yet been evaluated (31, 32).

In this study, an electrophysiological approach was used to biophysically and pharmacologically characterize BK channels in isolated muscle cells from the cricket lateral oviduct. We also investigated the functional linkage between L-type Ca^2+^ channels and BK channels. Our results show that Ca^2+^ influx via L-type Ca^2+^ channels induces the activation of nearby BK channels by functional coupling.

## Materials and Methods

### Insect Rearing

The sexually mature *Gryllus bimaculatus* females used in this study were purchased from a local pet store where they are sold as food for pet reptiles (i.e., genetic and environmental variability is limited). Crickets were housed in a covered plastic container with a shelter shaped from cardboard until required for use. All crickets were bred at 27 ± 2°C, in a 12 h light/dark cycle. Crickets had free access to feed and water for insects (I, Oriental Yeast Co., Ltd., Kyoto, Japan).

### Cell Isolation

Adult female crickets were fixed in the upper dorsal area under CO_2_ anesthesia. Lateral oviducts were exposed by removing the connective tissue around reproductive organs after a dorsal incision in the abdomen in normal saline (in mM): 140 NaCl, 10 KCl, 1.6 CaCl_2_, 2 MgCl_2_, 44 glucose, and 2 HEPES, pH adjusted to 7.4 with 2-amino-2-hydroxymethyl-1,3-propanediol(tris(hydroxymethyl)aminomethane) (Tris). Left and right lateral oviducts connected to the common oviduct from the vitellarium were excised. Enzymatic cell dissociation was performed using protease dispersion, as described previously (33). Isolated lateral oviduct myocytes were maintained in fresh saline at room temperature and used within 12 h.

### Electrophysiology

Cells were dropped onto a glass-bottom dish containing the experimental solution, and adhered cells were used for measurements. Cells were observed and imaged under an inverted microscope (IX70: OLYMPUS, Tokyo, Japan). Currents from cells were recorded at room temperature (22–27°C) using patch-clamp techniques using the cell-attached and excised inside-out modes, and an Axopatch 200B (Axon Instruments/Molecular Devices, Union City, CA, USA) or CEZ-2200 (Nihon Kohden, Tokyo, Japan) patch-clamp amplifier. Patch electrodes were prepared from capillary tubes (hemato-clad capillary, Drummond Scientific Co., Broomall, PA, USA) using a two-stage pipette puller (PC-10 Narishige, Tokyo, Japan). When filled with a solution for single-channel recordings, patch electrodes had a tip resistance of ~10 MΩ. Current signals were filtered at 5 kHz with a four-pole Bessel filter and digitized at 10 or 20 kHz. pCLAMP (version 6, 7, or 10; Axon Instruments/Molecular Devices) software was used for command pulse control, data acquisition, and analysis. The amplitude of single-channel currents and steady-state open probabilities (NPo) were determined by a cursor on Clampfit, Fetchan, or pStat or using the single-channel search mode of the pCLAMP software. Data were also analyzed using Origin software (OriginLab Corp., Northampton, MA, USA) and Sigma Plot (Systat Software, San Jose, CA, USA). For single-channel recordings, cell-attached recordings were obtained using normal saline as the external solution and a pipette solution that contained (in mM) 140 KCl, 10 NaCl, 1.6 CaCl_2_, 2 MgCl_2_, and 2 HEPES (pH adjusted to 7.4 by Tris). K^+^ selectivity of isolated myocytes was tested with, 100, 70, 35, and 10 mM KCl that was prepared by replacing the KCl in the pipette solution with an equal amount of NaCl (see Table 1). The Ca^2+^ dependence of BK channels was assessed by adding CaCl_2_ to the bath solution and adjusting the free Ca^2+^ concentration from 1 nM to 1 μM [calculated using CaBuf software (G. Droogmans, KU Leuven, Leuven, Belgium)]. The bath solution contained (in mM): 140 KCl, 10 NaCl, 5 EGTA, 2 MgCl_2_, and 2 HEPES (pH adjusted to 7.4 with Tris). The extracellular effects of iberiotoxin (IbTX) and tetraethylammonium (TEA) were tested by application in the pipette solution using the standard backfill method described previously (34). In brief, electrode tips were filled with normal pipette solution and then backfilled with the same solution containing the indicated concentration of the inhibitors being tested. Data were recorded after waiting for at least 10 min. Bay K8644 and nifedipine were dissolved in dimethylsulfoxide (DMSO) to create stock solutions, and aliquots were added to the perfusate. Concentrations of DMSO were below 0.1% in the treatment solution, and this had no observable effects on the cells. All activator and inhibitor reagents used were purchased from Sigma-Aldrich Corp. (St. Louis, MO, USA). All other reagents were purchased from Wako Pure Chemical Industries, Ltd. (Osaka, Japan).

**Table 1:** Composition of solutions (in mM).

	**Solution**	**NaCl**	**KCl**	**CaCl_**2**_**	**MgCl_**2**_**	**Glucose**	**HEPES**	**EGTA**
A	10 mM K^+^ (ringer)	140	10	1.6	2	44	2	0
B	35 mM K^+^	115	35	1.6	2	44	2	0
C	70 mM K^+^	80	70	1.6	2	44	2	0
D	105 mM K^+^	45	105	1.6	2	44	2	0
E	140 mM K^+^	10	140	1.6	2	44	2	0
F	Ca^2+^-free	140	10	0	3.6	44	2	0
G	Ca^2+^-free	140	10	0	0	44	2	5
H	1 nM Ca^2+^	140	10	0.08	0	44	2	5
I	10 nM Ca^2+^	140	10	0.67	0	44	2	5
J	100 nM Ca^2+^	140	10	3.05	0	44	2	5
K	1 μM Ca^2+^	140	10	4.7	0	44	2	5
L	10 μM Ca^2+^	140	10	4.98	0	44	2	5

^*^Adjusted to pH 7.4 with Tris-HCl.

### Statistical Analyses

All data are expressed as means ± standard error of the mean (SEM). Data for each condition were obtained from at least three independent experiments. A comparison of the means between groups was performed using unpaired Student's *t*-test and one-way ANOVA to assess statistical significance using Excel (Microsoft Corporation, Redmond, WA, USA) or Origin 8 (OriginLab Corp.) software. The data used for statistical analysis passed the Shapiro–Wilk normality test and the Levene equal variance test. For Figure 7F, the one-way ANOVA test used Levene's test for equal variance, and Bonferroni correction was used for the *post-hoc* test. *P* < 0.05 was considered statistically significant.

## Results

### Single-Channel Recording of BK Channels in Isolated Myocytes

**Figure 1:**
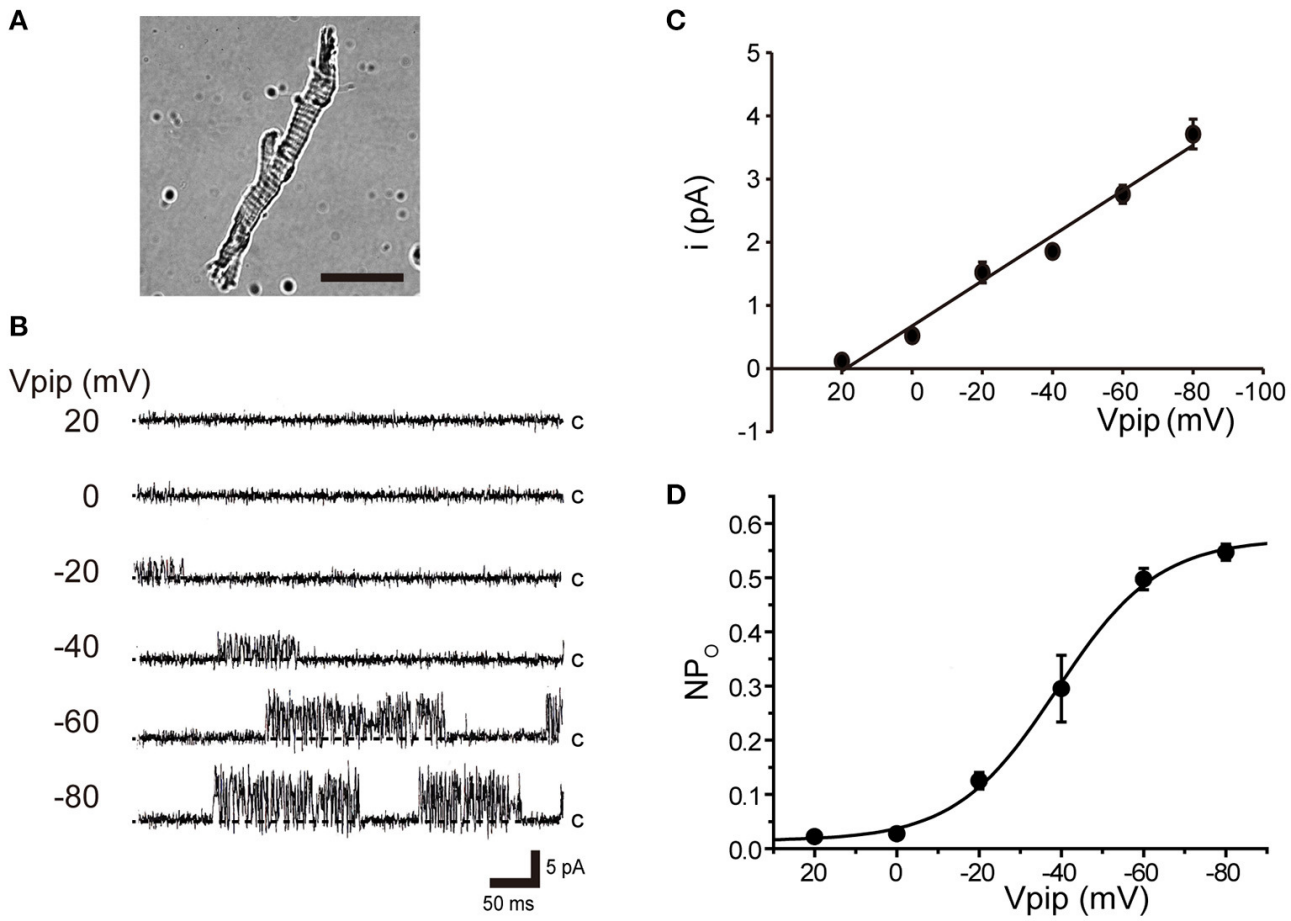
Voltage-sensitive single K^+^ channel current in isolated cricket oviduct cells. **(A)** A single myocyte isolated from the lateral oviduct by optical microscopy. The scale bar is 50 μm. **(B)** Single K^+^ channel current from cell-attached patches under an extracellular K^+^ concentration ([K^+^]_o_) of 10 mM. Representative single-channel current traces at various holding potentials (Vpip) are indicated in the figure. c indicates a closed level. **(C)** Averaged single-channel current (i) -Vpip (i-Vpip) relationship (n = 6–14). Data points from +20 mV to −80 mV of Vpip were fitted by linear regression to obtain a slope conductance of 35.7 pS. **(D)** The average number of channel and single-channel open probability (NPo)–Vpip relationship of steady-state single-channel current recorded at each Vpip. The NPo–Vpip curve fits the Boltzmann function with the voltage for half-maximal activation of −38.9 mV and the slope of 12.3 mV. A total of 59 cells isolated from a total of 75 animals were used in the experiment and 65 tests were performed for data collection.

**Figure 2:**
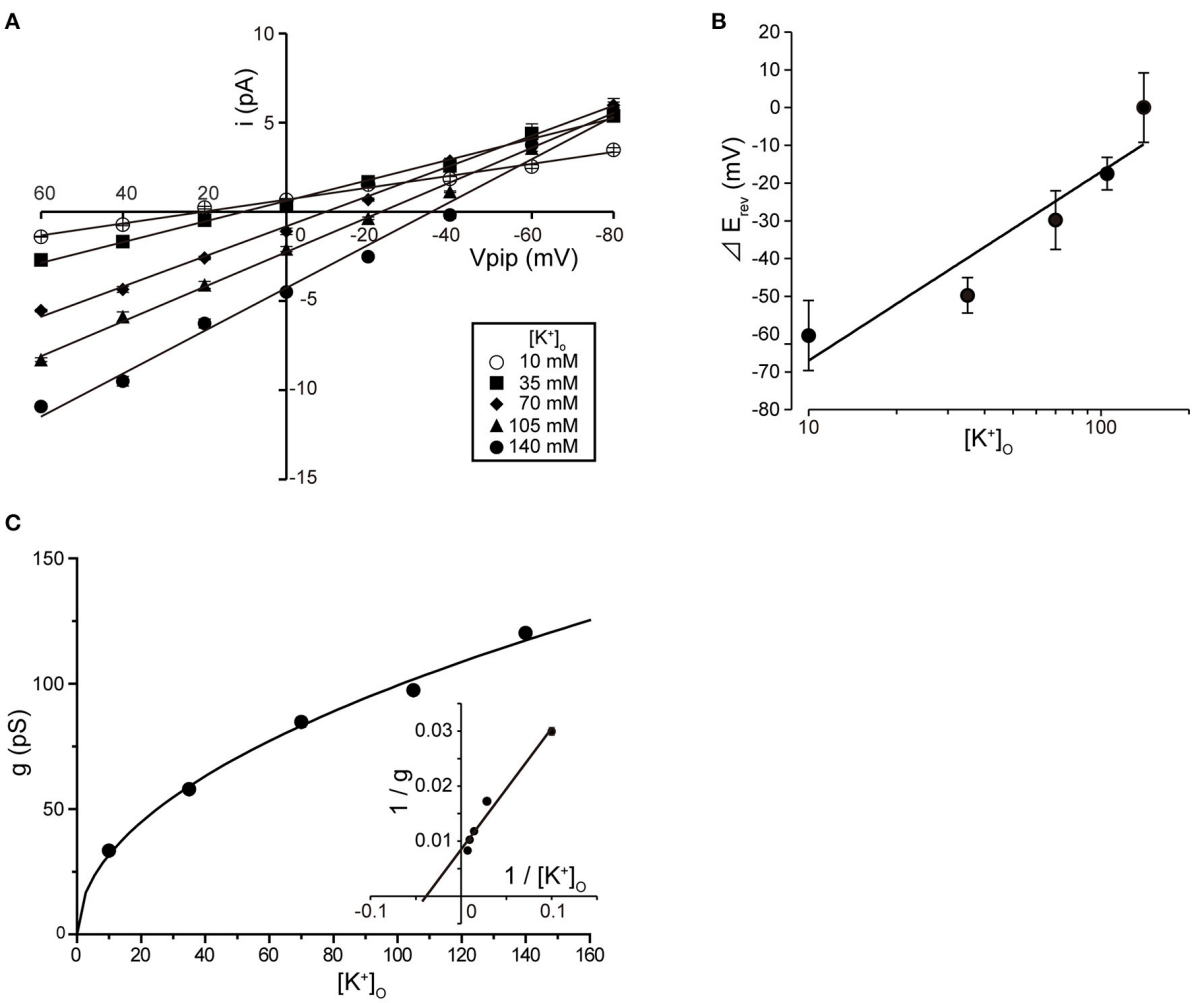
Single K^+^ channel IV relationship at various extracellular K^+^ concentrations. **(A)** Averaged IV data at various extracellular K^+^ concentrations ([K^+^]_o_), •, 140; ▲, 105; ◆, 70; ■, 35; ○, 10, from cell-attached patches. **(B)** The data show a semi-logarithmic plot of [K^+^]_o_ against the difference in reversal potential obtained at 140 mM and the reversal potential obtained at each K^+^ concentration (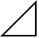
E_rev_). The E_rev_ was obtained by fitting the i–Vpip relationship of each K^+^ concentration in A by linear regression. The slope of the regression line shows 48.1 mV/decade. **(C)** The [K^+^]_o_ dependence of slope conductance (g) is shown. The value of each conductance was obtained by fitting the i–Vpip relationship of each K^+^ concentration of A by linear regression. The solid curve fits the Michaelis–Menten equation. The inset shows the same data plotted in double reciprocal format. g_max_, 117.6 pS; K_m_, 25.9 mM. A total of 56 cells isolated from a total of 46 animals were used in the experiment and 45 tests were performed for data collection.

**Figure 3:**
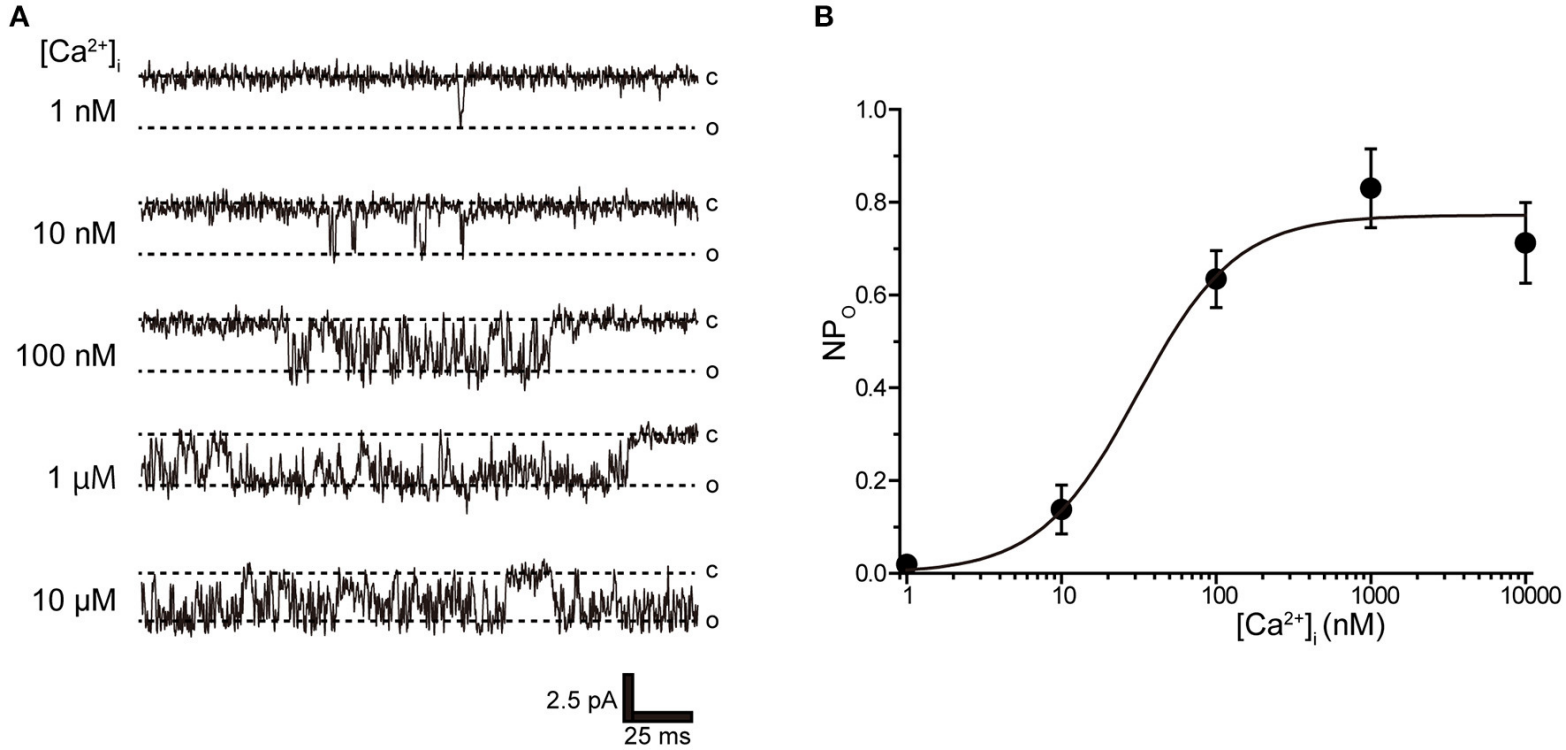
Intracellular Ca^2+^ concentration dependence of K^+^ channel activation. **(A)** Representative traces of single K^+^ channel currents recorded under various intracellular Ca^2+^ concentration ([Ca^2+^]_i_) conditions at Vpip = 0 mV from excised inside-out patches. c and o indicate closed and open levels, respectively. **(B)** Average NPo-[Ca^2+^]_i_ relationship of steady-state single-channel current. The data fit the Hill equation with an EC_50_ of 31.2 ± 10.6 nM and a Hill coefficient of 1.4. A total of 36 cells isolated from 32 animals were used in the experiment and 23 tests were performed to data collection.

Tubular oviductal myocytes have a distinct striped appearance with alternating bright and dark bands at regular intervals and a major axis of 100 μm and a minor axis of 5 μm (Figure 1A). Single-channel recording was used to eliminate the effects of muscle contraction and resting membrane potential using a previously reported extracellular Ca^2+^-free high-concentration K^+^ solution (33, 35). A previous report revealed no single K^+^ channel current in the extracellular solution used (35). In this study, we used a physiological solution containing Ca^2+^ to produce and observe single-current channels. When the holding potential was maintained at the depolarizing potential in cell-attached mode, the single-channel opening was consistently observed in the current amplitude type with burst-like kinetics (Figures 1B, 2). Single-channel currents recorded from +20 mV to −80 mV of the holding potential showed a linear current–voltage (IV) curve with a slope conductance of 35.7 pS with respect to voltage (*n* = 6–14) (Figure 1C). NPo increased with increasing depolarizing potential (Figure 1D). We then investigated the effect on single-channel conductance and reversal potential by altering extracellular K^+^ concentrations (Solutions A–E in Table 1). The IV relationship constructed from single-channel recordings at holding potentials of +20 mV to −80 mV (Figure 2A) shows a linear IV relationship under the conditions of five different extracellular K^+^ concentrations. Assessment of linear IV relationships using least-square analysis showed reverse potentials of 20.6, 10.3, −9.7, −23.1, and −35.5 mV for channel currents recorded under 10, 35, 70, 105, and 140 mM conditions, respectively. The slope of the E_rev_ change for extracellular K^+^ concentration changes was 48.1 mV per decade change in K^+^ concentration (Figure 2B). Conversely, the slope conductance of the channel obtained from the linear approximation fitting in Figure 2A increased with increased extracellular K^+^ (Figure 2C). The extracellular K^+^ concentration dependence of conductance was estimated as 120 ± 11.7 pS for maximum channel conductance and 26.4 ± 5.8 mM for Km by fitting a single Michaelis–Menten equation (*n* = 5–7). BK channels have the unique property of being activated by increased [Ca^2+^]_i_, in addition to being voltage-gated and having high conductance (1, 2). To directly assess the dependence of K^+^ channels on [Ca^2+^]_i_, single-channel currents were measured from inside-out patches of excised membranes exposed to a bath solution containing various concentrations of Ca^2+^ (Solutions G–L in Table 1, Figure 3). The NPo of K^+^ currents recorded at various concentrations in the Ca^2+^ bath at a potential of 0 mV became more frequent with increasing [Ca^2+^]_i_ in a manner suitable for the Hill equation with a k value of 31.2 μM and a Hill coefficient of 1.4.

### Pharmacology of BK Channels

**Figure 4:**
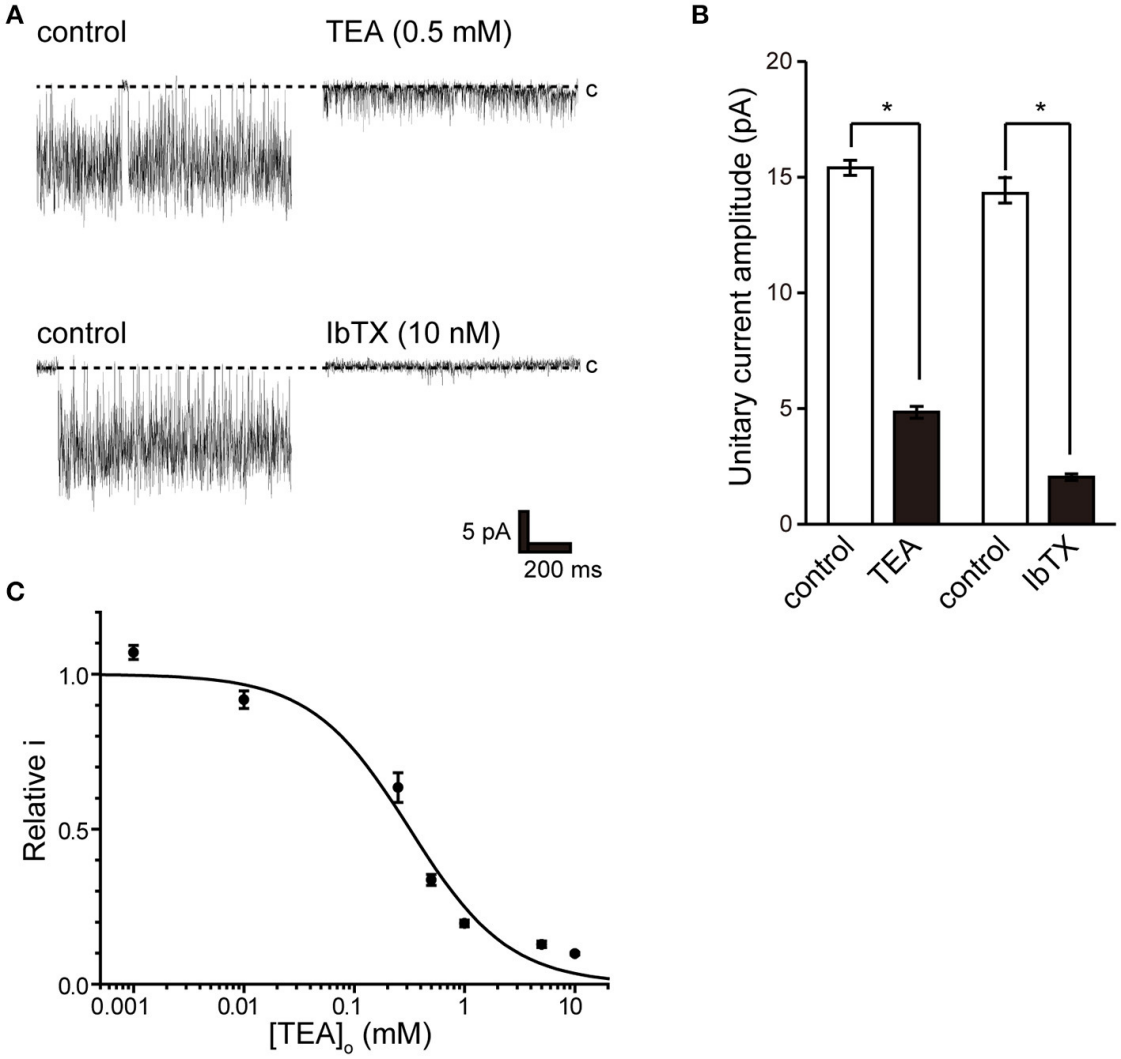
Effect of tetraethylammonium and iberiotoxin on K^+^ channel current. **(A)** (top) Single-channel current in the absence (control) and presence of extracellular 0.5 mM tetraethylammonium (TEA) using the backfill method. (lower) Single-channel current in the absence (control) and presence of 10 nM extracellular iberiotoxin (IbTX) using the backfill method. The Vpip was 100 mV. c indicates a closed level. **(B)** The averaged amplitude of the single-channel currents in the absence and presence of TEA (*t*-value 25.16) or IbTX (*t*-value 25.16), respectively. ^*^Significantly different (*P* < 0.05) from control values. **(C)** Average relative relationship in the presence of TEA concentration ([TEA]_o_) to the control value of single K^+^ channel current amplitude. The data fit the Hill equation with an IC_50_ of 318.8 ± 68.8 μM and a Hill coefficient of 1.0. A total of 83 cells isolated from 11 animals were used in the experiment, and 11 tests were performed for data collection.

To pharmacologically characterize BK channels, the effects of the established inhibitor tetraethylammonium (TEA) (36) and the scorpion-derived IbTX peptide (37, 38) on channel currents were assessed. The backfill method (33) was used to test the inhibitory effect of BK channel blockers from the outside of the cell. The inhibitory effect on the endogenously expressed BK channel was evaluated by the magnitude of the single-channel amplitude that appeared more than 5 min after backfilling. Consistent BK channel currents were recorded for each measurement immediately after measuring in cell-attached mode (left trace of Figure 4A). After 10 min, both TEA and IbTX inhibited single-channel current amplitude (Figure 4A right trace, Figure 4B). Consistent with previous reports (9), the effect of TEA shows open-channel blocking behavior (Figure 4A), allowing a stable evaluation of single-channel current amplitude. Extracellular TEA administration revealed a concentration-dependent inhibitory effect on the current amplitude of a single BK channel with an IC_50_ value of 318.8 ± 68.8 μM (Figure 4C).

Together, the biophysical properties, [Ca^2+^]_i_ dependence, and pharmacological properties show that the recorded single-channel current is a BK channel that is endogenously expressed in isolated myocytes.

### Functional Coupling Between BK and Ca^2+^ Sources

**Figure 5:**
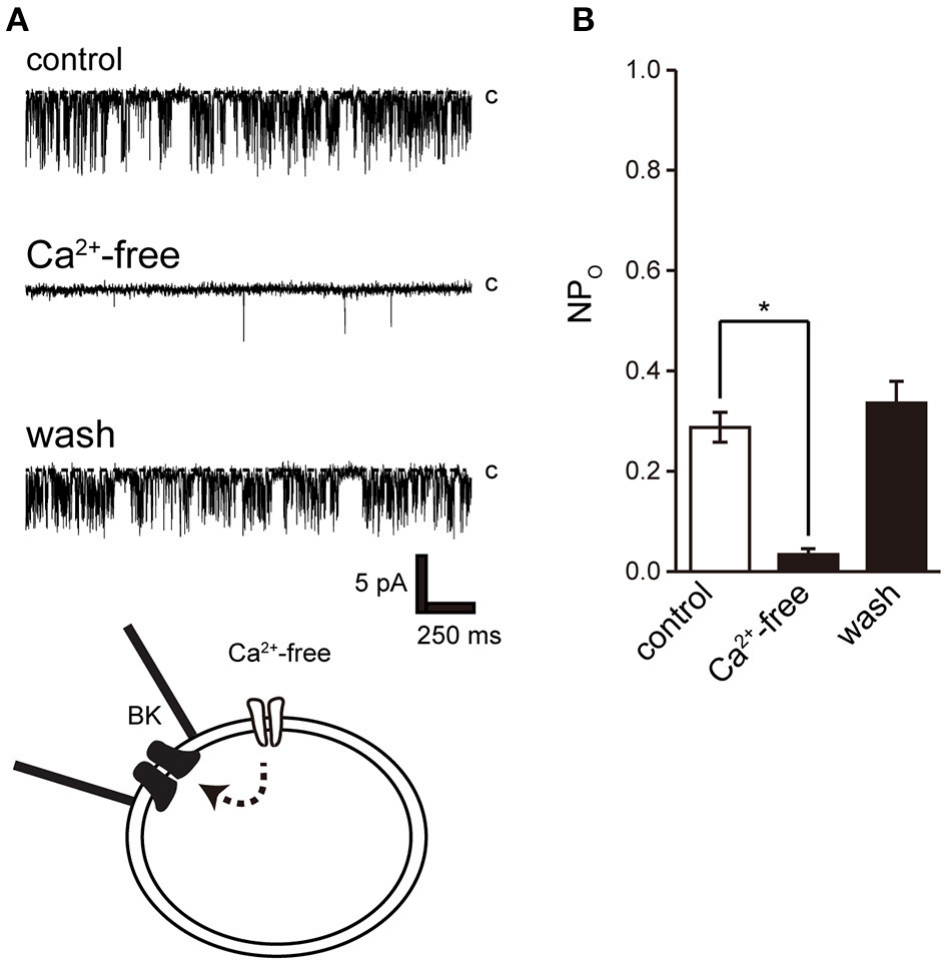
Effect of Ca^2+^ removal from bath solution on BK channel activity. **(A)** Representative BK channel currents before (control) and after Ca^2+^ removal from bath solution (Ca^2+^-free; Solution F in Table 1) and after washout (washout). The current was recorded from cell-attached patches. c indicates a closed level. **(B)** Average NPo of BK channel current. Recordings were made at a holding potential of −10 mV. ^*^Significantly different (*P* < 0.05) from control values (*t*-value 25.16). A total of 18 cells isolated from 15 animals were used in the experiment and 14 tests were performed for data collection.

Activation of Ca^2+^-dependent BK channels in excitatory cells is important for feedback control of Ca^2+^ influx and cell excitability. To examine the effect of Ca^2+^ on BK channel activity, we removed extracellular Ca^2+^. Extracellular Ca^2+^ removal resulted in suppression of BK activity after consistent BK channel activity (Figure 5).

We then investigated the pathways of intracellular Ca^2+^ release from intracellular Ca^2+^ stores and extracellular Ca^2+^ influx and their effect on BK activity. Ryanodine receptors play a central role in Ca^2+^ release in muscle cells, including skeletal muscle and myocardium with clear striated muscle (Figure 1A) (39). Furthermore, ryanodine receptors are involved in contraction in cricket lateral oviduct cells (28). L-type Ca^2+^ channels are another Ca^2+^ source and are functionally expressed in cricket lateral oviduct cells (35).

Ryanodine and caffeine are ryanodine receptor inhibitors and suppress constitutive BK activity under the same experimental conditions as were used in the previous experiment. Nifedipine administration decreased BK activity, whereas Bay K 8644 administration increased BK activity (Figures 7A–C). We investigated the proximity of BK and L-type Ca^2+^ channels within the microdomain by administering Bay K 8644 through a patch pipette and observing the effect on BK activity on the patch membrane. By recording with the holding voltage maintained at 0 and −60 mV, the effects of resting membrane potential and depolarization on L-type Ca^2+^ channel activity were investigated. BK activity was suppressed at −60 mV rather than at 0 mV (Figures 7E,F control). In all measurements, administration of Bay K 8644 increased BK activity only at 0 mV (Figures 7E,F).

## Discussion

**Figure 6:**
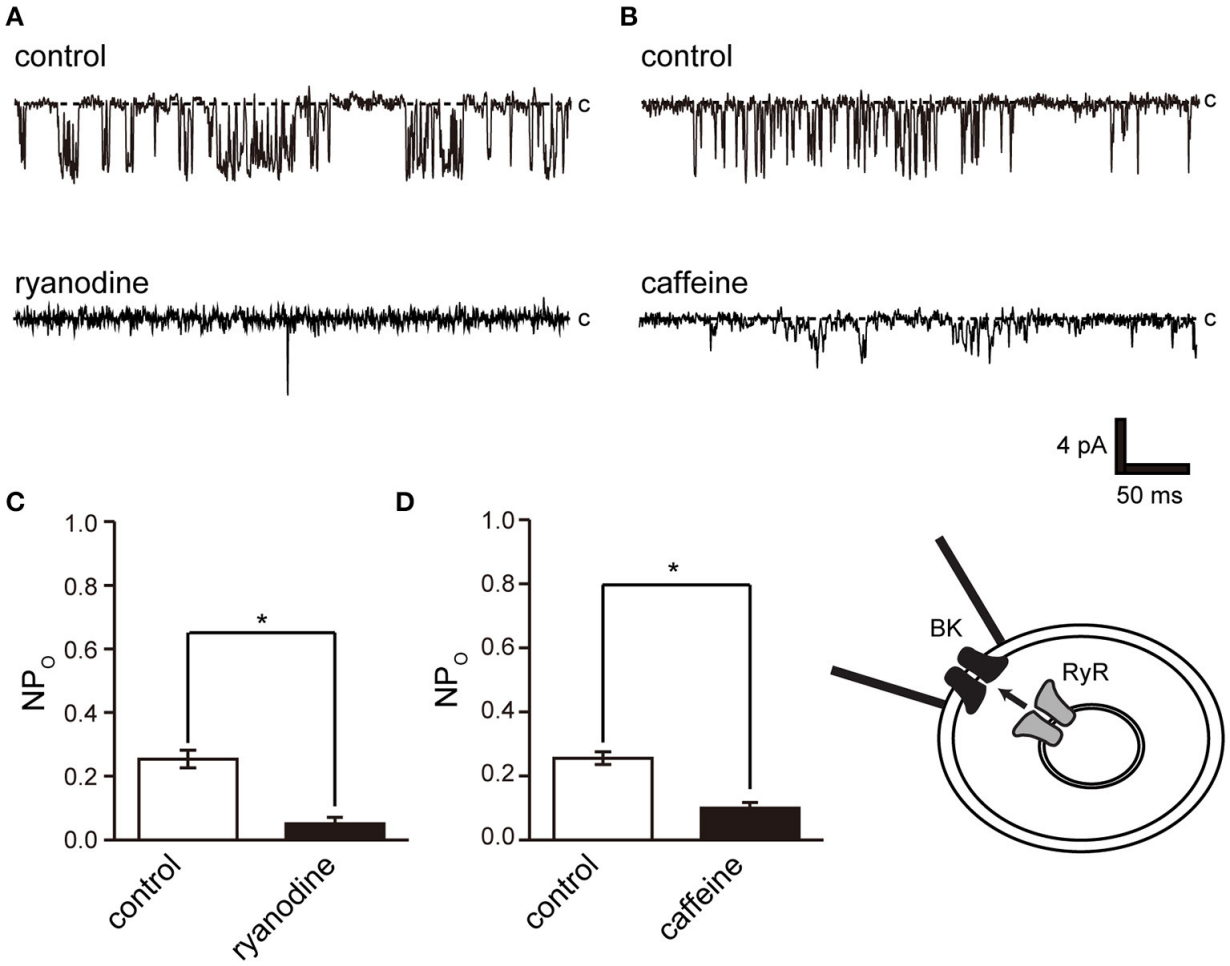
Effect of ryanodine and caffeine on BK channel activity. **(A)** Representative BK channel currents before (control) and after addition of 40 μM ryanodine (ryanodine) to the bath solution. c indicates a closed level. **(B)** Representative BK channel currents before (control) and after addition of 10 mM caffeine (caffeine) to the bath solution. c indicates a closed level. **(C,D)** Average NPo of BK channel current. Recordings were made at a holding potential of −10 mV. ^*^Significantly different (*P* < 0.05) from control values (*t*-values, ryanodine: 5.91, caffeine: 5.80). A total of 18 cells isolated from a total of 12 animals were used in the experiment, and 12 tests were performed for data collection.

**Figure 7:**
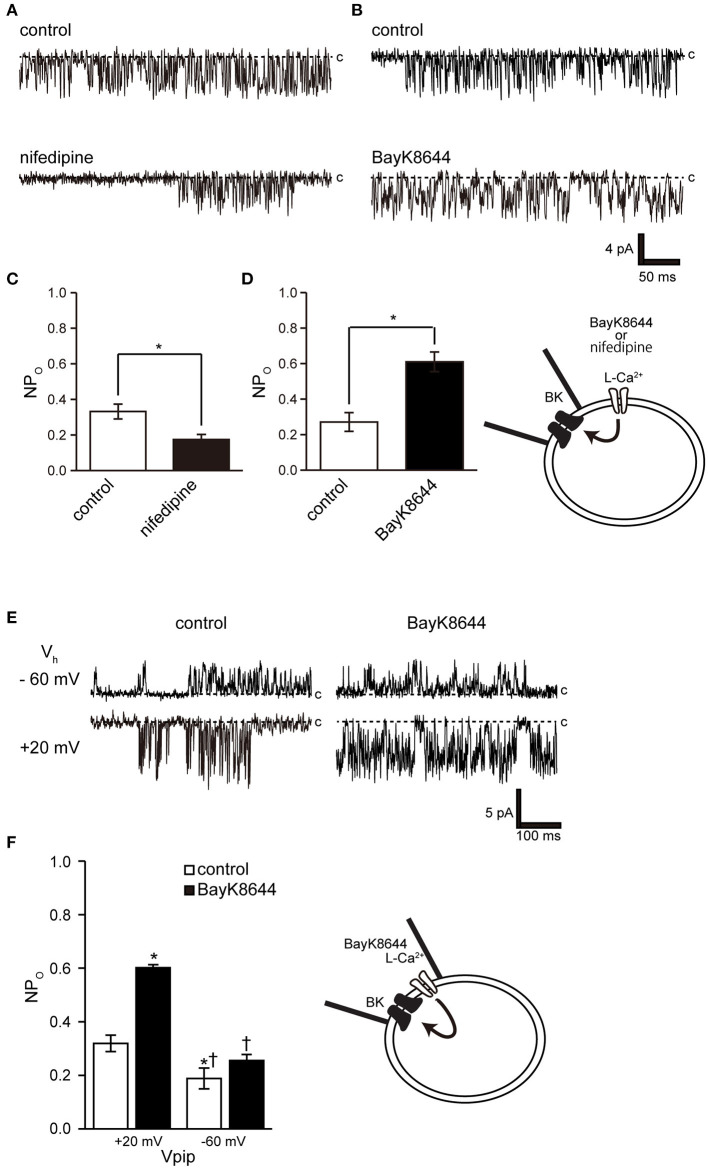
Effect of nifedipine and Bay K 8644 on BK channel activity. **(A)** Representative BK channel currents before (control) and after addition of 5 μM nifedipine (nifedipine) to the bath solution. c indicates a closed level. **(B)** Representative BK channel currents before (control) and after addition of 5 μM S(-)-Bay K 8644 (BayK8644) to the bath solution. The current was recorded at a Vpip of −10 mV from cell-attached patches. c indicates a closed level. **(C,D)** Average NPo of BK channel current (*t*-values, nifedipine: 3.13, BayK8644: 3.13). **(E)** Representative BK channel currents before (control) and after addition of extracellular 5 μM S(-)-Bay K 8644 (BayK8644) using the backfill method at a Vpip of −60 and 0 mV, respectively, from cell-attached patches. c indicates a closed level. **(F)** Average NPo of BK channel current. ^*^Significantly different (*P* < 0.05) from control at +20 mV values.^†^Significantly different (*P* < 0.05) from BayK8644 at +20 mV values. A total of 39 cells isolated from a total of 32 animals were used in the experiment, and 25 tests were performed for data collection.

In this study, we performed patch-clamp electrophysiology to characterize the functional expression of BK channels in cricket oviduct cells. We demonstrated, for the first time, that BK channels are endogenously expressed in this cell type. A series of recordings of single-channel activity and the appropriate classification criteria, such as K^+^ selectivity, high conductance dependent on extracellular K^+^ concentration, voltage dependence, and intracellular Ca^2+^ sensitivity, revealed these channels as BK channels (Figures 1–3). Extracellular application of TEA and IbTX inhibited single-channel current amplitude (Figure 4). The inhibition sensitivity of TEA on cricket BK channels (IC_50_ = 318.8 μM) is within the range observed in humans and *Drosophila* (IC_50_ = 80–330 μM) (40). These properties are consistent with those of high-conductance vertebrate BK channels (1, 2). The BK channel considered representative of invertebrate BK channels is that of the *Drosophila slo* family (41, 42), which has common properties across species, including crayfish (43) and locust (44, 45). Examination of the Ca^2+^ source that activates BK channels revealed that extracellular Ca^2+^ influx was essential for BK channel activity (Figure 5). We also observed that the effects of Ca^2+^ influx and release by L-type Ca^2+^ channel regulators and ryanodine receptor regulators affect BK activity (Figures 6, 7A–D). The effect of Bay K 8644 in the patch pipette indicated the proximity of BK and L-type Ca^2+^ channels in the microregions of the patch membrane (Figures 7E,F).

The activation threshold of BK channels at resting membrane potential reflects the associated voltage-gated Ca^2+^ activation threshold because, at this potential, it is mainly activated by [Ca^2+^]_i_. Dihydropyridine-sensitive L-type Ca_v_1.1–1.4 exhibit unique activation threshold profiles. Ca_v_1.3 and 1.4 can be activated at low voltage thresholds, and Ca_v_1.3 can initiate activation at a negative voltage of −55 mV (46). Accordingly, it activates BK at a low potential (near −50 mV) in neuronal cells (19). Cricket myocytes exhibit voltage-gated Ca^2+^ channel currents with properties similar to those of Ca_v_1.2, which are activated at around −40 mV with a peak at 0 mV (35, 46, 47). These results indicate that it is unlikely that Ca^2+^ influx through the activity of L-type Ca^2+^ channels can be expected in cells with a resting membrane potential near −50 mV. However, the resting membrane potential of cricket myocytes was more comparable to that previously reported [−25.8 ± 2.8 mV (*n* = 9)] and to Numata's unpublished observation [−24 ± 2.5 mV (47)]. Therefore, the BK channel in this experiment is likely to be activated even though L-type Ca^2+^ channel activity exceeded the threshold value of around −25 mV. BK activity was controlled by the dihydropyridine receptor modulator at the resting membrane potential (Figures 7A–D), but Bay K 8644 administration had no effect at depolarizing potentials, causing the L-type Ca^2+^ channel to enter a steady inactivated state (Figures 7E,F). The physiological importance of BK activity at the relatively low resting membrane potentials obtained in this experiment may also apply to species exhibiting similar resting membrane potentials, including *Drosophila* muscle [−40 mV (48)], earthworm [−37 mV (49)], *C. elegans* [−20 to −25 mV (50, 51)], and *Ascaris lumbricoides* [−33 mV (52)].

Opening a single Ca^2+^ channel increases local [Ca^2+^] to over 100 μM within tens of nanometers of the inner mouth of the channel, but most of these ions are buffered within microseconds (53). The BK channel is found in close proximity to all Ca^2+^ channel subfamilies on the cell membrane (16, 54–56). This association is reproducible in heterogeneous systems and when reconstituting functional nanodomains (19, 57). The key to establishing this nanodomain is the high Ca^2+^ sensitivity of BK channels (EC_50_ = 10 nM−1 μM) (2) and the proximity to Ca^2+^ channels (57). Our results show that cricket muscle cell BK channels have an intracellular Ca^2+^ sensitivity of EC_50_ of 31.2 nM. This shows that they are highly sensitive to intracellular Ca^2+^ and is similar that of invertebrate locust muscle (100 pM−1 nM) (45). Furthermore, cell-attached mode measurements within 1 μm of the patch pipette show the proximity of the L-type Ca^2+^ channel to the BK channel (Figures 7E,F). These observations show that the BK channel has sufficient properties for the construction of a microdomain in this study. Further detailed studies require molecular identification of cricket ion channels and clarification of the functional and positional relationships between BK channels and Ca^2+^ sources in cells.

In conclusion, characterization of a single BK channel in cricket oviduct cells provides a model for investigating the functional association with Ca^2+^ sources. The BK channel characterized in this study was activated near the resting membrane potential by functional coupling with a voltage-gated Ca^2+^ channel with spontaneous activity. We propose that cricket muscle cells are involved in spontaneous contraction (28) via microdomains of BK channels and L-type Ca^2+^ channels.

## Data Availability Statement

The original contributions presented in the study are included in the article/supplementary material, further inquiries can be directed to the corresponding author/s.

## Author Contributions

TN and MY: conceptualization, study design, and funding acquisition. TN and KS-N: performance, formal analysis, writing, revision, and editing. TN: writing of the original draft. MY: supervision. All authors contributed to the article and approved the submitted version.

## Conflict of Interest

The authors declare that the research was conducted in the absence of any commercial or financial relationships that could be construed as a potential conflict of interest.
